# Establishment of an ovarian cancer omentum metastasis-related prognostic model by integrated analysis of scRNA-seq and bulk RNA-seq

**DOI:** 10.1186/s13048-022-01059-0

**Published:** 2022-11-23

**Authors:** Dongni Zhang, Wenping Lu, Shasha Cui, Heting Mei, Xiaoqing Wu, Zhili Zhuo

**Affiliations:** Oncology Department, China Academy of Chinese Medical Sciences Guang’anmen Hospital, Beijing, China

**Keywords:** Ovarian cancer, scRNA-seq, Omentum metastasis, 6-OMAGs, Prognosis, Tumor microenvironment

## Abstract

**Objective:**

Ovarian cancer has the highest mortality rate among gynecological malignant tumors, and it preferentially metastasizes to omental tissue, leading to intestinal obstruction and death. scRNA-seq is a powerful technique to reveal tumor heterogeneity. Analyzing omentum metastasis of ovarian cancer at the single-cell level may be more conducive to exploring and understanding omentum metastasis and prognosis of ovarian cancer at the cellular function and genetic levels.

**Methods:**

The omentum metastasis site scRNA-seq data of GSE147082 were acquired from the GEO (Gene Expression Omnibus) database, and single cells were clustered by the Seruat package and annotated by the SingleR package. Cell differentiation trajectories were reconstructed through the monocle package. The ovarian cancer microarray data of GSE132342 were downloaded from GEO and were clustered by using the ConsensusClusterPlus package into omentum metastasis-associated clusters according to the marker genes gained from single-cell differentiation trajectory analysis. The tumor microenvironment (TME) and immune infiltration differences between clusters were analyzed by the estimate and CIBERSORT packages. The expression matrix of genes used to cluster GSE132342 patients was extracted from bulk RNA-seq data of TCGA-OV (The Cancer Genome Atlas ovarian cancer), and least absolute shrinkage and selection operator (LASSO) and multivariate Cox regression were performed to establish an omentum metastasis-associated gene (OMAG) signature. The signature was then tested by GSE132342 data. Finally, the clinicopathological characteristics of TCGA-OV were screened by univariate and multivariate Cox regression analysis to draw the nomogram.

**Results:**

A total of 9885 cells from 6 patients were clustered into 18 cell clusters and annotated into 14 cell types. Reconstruction of differentiation trajectories divided the cells into 5 branches, and a total of 781 cell trajectory-related characteristic genes were obtained. A total of 3769 patients in GSE132342 were subtyped into 3 clusters by 74 cell trajectory-related characteristic genes. Kaplan-Meier (K-M) survival analysis showed that the prognosis of cluster 2 was the worst, *P* < 0.001. The TME analysis showed that the ESTIMATE score and stromal score in cluster 2 were significantly higher than those in the other two clusters, *P* < 0.001. The immune infiltration analysis showed differences in the fraction of 8 immune cells among the 3 clusters, *P* < 0.05. The expression data of 74 genes used for GEO clustering were extracted from 379 patients in TCGA-OV, and combined with survival information, 10 candidates for OMAGs were filtered by LASSO. By using multivariate Cox regression, the 6-OMAGs signature was established as RiskScore = 0.307*TIMP3 + 3.516*FBN1–0.109*IGKC + 0.209*RPL21 + 0.870*UCHL1 + 0.365*RARRES1. Taking TCGA-OV as the training set and GSE132342 as the test set, receiver operating characteristic (ROC) curves were drawn to verify the prognostic value of 6-OMAGs. Screened by univariate and multivariate Cox regression analysis, 3 (age, cancer status, primary therapy outcome) of 5 clinicopathological characteristics were used to construct the nomogram combined with risk score.

**Conclusion:**

We constructed an ovarian cancer prognostic model related to omentum metastasis composed of 6-OMAGs and 3 clinicopathological features and analyzed the potential mechanism of these 6-OMAGs in ovarian cancer omental metastasis.

**Supplementary Information:**

The online version contains supplementary material available at 10.1186/s13048-022-01059-0.

## Introduction

Ovarian cancer has the highest mortality rate among gynecological malignant tumors [1]. At present, no effective method for the early detection of ovarian cancer has been found. 70% of clinical ovarian cancer is advanced at the time of diagnosis, while after surgery and chemotherapy, 70% of patients will still have metastasis within 2–3 years [2]. Ovarian cancer metastasis has distinct characteristics: it preferentially metastasizes to omental adipose tissue, leading to intestinal obstruction and death [3]. However, the mechanism of this tropism remains unclear. Finding the cause of this transfer and preventing it has been a crucial problem that researchers hope to solve for many years.

The omentum is a special adipose tissue in the peritoneal cavity that is composed of adipocytes and stromal blood vessels, comprising preadipocytes, fibroblasts, vascular endothelial cells and a variety of immune cells that can promote various immune responses, including inflammation, tolerance and fibrosis, thereby promoting peritoneal immunity [4]. Studies have found that after the initial cytoreductive surgery, there are still residual latent cancer cells [5]. The production of substances such as inflammatory factors may lead to the migration/invasion of latent cancer cells into the omentum, resulting in changes in the omental microenvironment. Epithelial mesenchymal transformation (EMT), angiogenesis, immune infiltration, inflammation, etc. [6–9] drive the formation of a niche before metastasis and contribute to successful transmission, that is, the premetastatic microenvironment (PMN). It has been demonstrated that circulating tumor cells (CTCs) exist in the blood of ovarian cancer patients, and hematogenous metastasis can be a crucial mode of omentum metastasis [3]. When tumor cells are planted in the omentum, they can support tumor growth through immune and metabolic mechanisms [8]. The interaction between latent cancer cells and the PMN may be decisive for the metastasis program [10].

Single-cell RNA sequencing (scRNA-seq) uses optimized next-generation sequencing technology to define the global gene expression profile of single cells, which is helpful to isolate the previously hidden heterogeneity in the cell population, especially for the study of tumor heterogeneity [11]. Therefore, exploring the microenvironment of omental ovarian cancer at the single-cell level may be a feasible strategy to find the reasons for this tendency of metastasis. What characteristics of the omentum microenvironment are easily colonized by tumor cells, and what characteristics of ovarian cancer cells will be more prone to metastasis and invasion, furthermore, can these characteristics be used for the prognosis of clinical ovarian cancer patients. These are the questions that this study attempts to answer.

Recently, by using scRNA-seq from ovarian tumors resected from omental metastases, Olalekan S et al. [12] focused on T cell infiltration and performed remarkable work in revealing immune cell types and subsets with possible roles in the management of disease, suggesting an antitumor response in high T cell infiltration patients. Liu C et al. [13] used the scRNA-seq data of the former, combined it with the bulk RNA-seq data of TCGA-OV, identified four M2 tumor-associated macrophage (TAM)-associated genes with prognostic value in ovarian cancer patients and validated them by experiments. Their research is undoubtedly valuable but mainly focuses on certain types of immune cells. Millstein J. et al. [14] selected 200 genes associated with overall survival (OS) from a meta-analysis of six transcriptome-wide microarray studies and 313 genes based on the literature and unpublished data. They finally determined 276 genes associated with OS by using 2702 tumors from 15 studies and evaluated 1067 tumors from six studies. This discovery provides a fabulous opportunity for the development of targeted therapeutic approaches; however, the specific mechanism of these genes in the progression of ovarian cancer and the correlation between them need to be further clarified.

Here, we used the scRNA-seq data of Olalekan S’s study to explore significantly differentially expressed genes during the differentiation trajectories of ovarian cancer omentum metastasis sites. According to the expression levels of these genes, 3769 ovarian cancer patients from Millstein J.’s study (GSE132342) were grouped to determine their relationship with clinical characteristics, physiological relevance with omentum metastasis and prognosis. These genes were further used to screen ovarian cancer omentum metastasis-related prognostic genes by using bulk RNA-seq data in TCGA-OV and verified with patients in GSE132342. The results might provide insight into the molecular mechanism and characteristics of ovarian cancer omentum metastasis and its connection with patient survival.

## Materials and methods

### Data acquisition

**Fig. 1:**
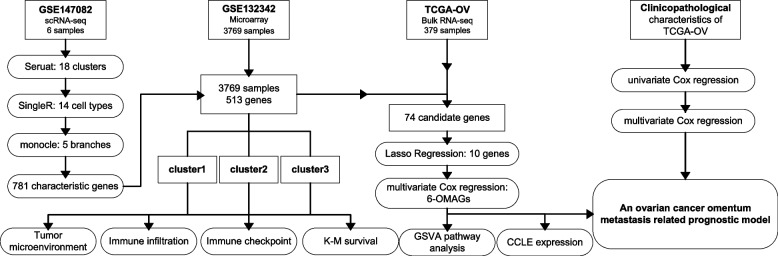
The technical workflow

The scRNA-seq data were downloaded from the Gene Expression Omnibus (GEO, https://www.ncbi.nlm.nih.gov/geo/) database, accession number GSE147082, including 9,885 cells and 16,041 genes isolated from the omental metastatic site of 6 ovarian cancer patients. The bulk RNA-seq data of 379 ovarian cancer patients and 56,461 genes in TCGA-OV were obtained from The Cancer Genome Atlas (TCGA, https://portal.gdc.cancer.gov/) database. The microarray data were acquired from GEO with accession number GSE13234, which contained 3769 ovarian cancer patients of 513 genes (Fig. 1).

### scRNA-seq data processing and analysis

By using the R package Seurat [15] (version 4.0.6), the scRNA-seq data were converted into a Seurat object by the function CreateSeuratObject(), while the PercentageFeatureSet() function was used to calculate the percentage of mitochondrial genes. The raw data were already filtered by the uploader by the criteria of rejecting unqualified data: a minimum cutoff of 600 genes per cell was set, and 7.8% counts of mitochondrial origin per cell; therefore, further filtering was not necessary [12]. The data were then normalized with the method “LogNormalize”. The top 1500 genes with a large coefficient of variation between cells were extracted by the function FindVariableFeatures(). Dimheatmap, JackStrawPlot and ElbowPlot were used to define the most significant principal component (PC) value for cell clustering. Subsequently, cell cluster classification was assessed by t-SNE (t-Distributed Stochastic Neighbor Embedding), and the maker genes of each cluster were screened by the function FindAllMarkers() with the cutoff values of |log2 fold change(FC)| > 1, the expression ratio of cell population > 0.25 and adjusted *P*-value < 0.05. SingleR [16] (Version: 1.8.0) package was utilized for cell cluster annotation, while the reference was loaded from the celldex package HumanPrimaryCellAtlasData() and combined with marker genes from the literature [17–20]. Cell differentiation trajectories were reconstructed through the monocle [21] (Version: 2.22.0) package. Based on marker genes that differed between clusters, dimension reduction was conducted by the reduceDimension function with reduction_method = “DDRTree” and max_components = 2. Characteristic genes of different cell states for downstream analysis were filtered by the criteria of |log2FC| > 1 and adjusted *P*-value < 0.05.

### GEO microarray data processing and analysis

The microarray data downloaded from GSE132342 were already log2 transformed, and the data were then standardized by using the normalizeBetweenArrays() function in the limma [22] (Version: 3.50.0) package. The characteristic genes of the different cell states above were used for clustering the patients in GSE132342 via the ConsensusClusterPlus [23] (Version: 1.58.0) package, with the specific parameters maxK = 6, reps = 50, pItem = 0.8, pFeature = 1, distance="euclidean”, and clusterAlg="pam“ [24]. The difference in survival among clusters of patients was analyzed by Kaplan-Meier (K-M) survival curve.

### Tumor microenvironment analysis

In the tumor microenvironment (TME), immune cells and stromal cells are two main types of normal cells. By using the expression data, an estimate algorithm can predict the score of stromal cells and immune cells, and the tumor purity in each tumor sample can then be calculated [25]. The ESTIMATE score, immune score, stromal score, and tumor purity of different clusters of patients from GSE132342 were calculated by using the estimate (https://bioinformatics.mdanderson.org/estimate/rpackage.html) R package.

### Immune infiltration analysis

CIBERSORT is an analytical tool developed by Newman et al. [26] to provide an estimation of the abundances of member cell types in a mixed cell population. With the R package “e1071” (Version: 1.7-9) loaded as a precondition, CIBERSORT was used to estimate the different abundances of 22 immune cells among different clusters of GSE132342.

### Immune checkpoint analysis

Several important immune checkpoints related to cancer were analyzed by the limma [22] (Version: 3.50.0) package. Differentially expressed immune checkpoint-related genes significantly associated with overall survival (OS) in clustered GSE132342 patients were determined by K-M survival analysis.

### Construction of a nomogram model in accordance with the OMAG risk signature

The characteristic genes of different cell states used for clustering the patients in GSE132342 were taken as the candidate genes for omentum metastasis-associated genes (OMAGs). The glmnet [27] (Version: 4.1-3) and survival (Version: 3.2–13) R packages were used to perform LASSO Cox regression analysis and 10-fold cross-validation to narrow the range of OMAG candidate genes. The OMAG candidates were then screened again by Cox multivariate regression analysis to determine the ultimate OMAG risk model. The formula of the OMAG signature was as follows: Risk score = Ʃ (βi x Expi), where βi represented the coefficient of gene i, standing for the weight of gene i, and Expi represented the expression level of gene i. Then, the TCGA-OV dataset was set as the training set, while GSE132342 was used as the test set. The effects of high- and low-risk scores on survival and the prognostic value of the OMAG risk signature were evaluated. The clinicopathological features and risk score of the TCGA-OV cohort were analyzed by univariate Cox regression and multivariate Cox regression using the survival (Version: 3.2–13) R package, with *p* < 0.05 as the criterion, and the HR and regression coefficient for each prognostic feature were calculated. Finally, the rms (Version: 6.2-0) R package was used to construct a nomogram to predict the OS of ovarian cancer patients, which incorporated these clinicopathological features and OMAGs. The nomogram model was a prognostic statistical model made using simple graphs according to previous studies [28].

### Correlation analysis between 6-OMAGs and tumor-related pathways

By using the GSVA [29] R package, the parameter was chosen as method=’ssgsea’. The correlation between 6-OMAGs and 20 tumor-related pathway scores in TCGA-OV was analyzed by Spearman correlation [30]. A *P*-value < 0.05 and ρSpearman > 0.3 were considered statistically significant.

### Expression analysis of 6-OMAGs in 45 human ovarian cancer cell lines

The mRNA expression matrix of 45 human ovarian cancer cell lines were obtained from the CCLE dataset (https://portals.broadinstitute.org/ccle) [31]. The analysis was constructed by the ggplot2 (version: 3.3.3) R package.

### Statistical analysis

All the data were processed and analyzed by using Perl and R software (version: 4.1.2). Comparisons of clinicopathological characteristics were performed through Wilcoxon rank-sum tests for quantitative variables and chi-square or Fisher’s exact tests for categorical variables. Differences among multiple groups were analyzed by Kruskal–Wallis’s test.

## Results

### scRNA-seq data processing and analysis

**Fig. 2:**
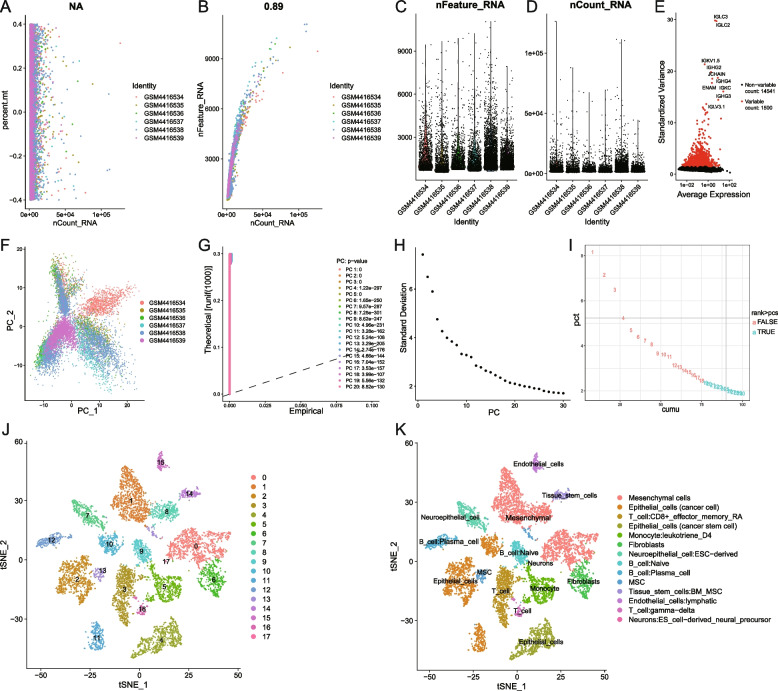
scRNA-seq data processing and analysis. **A** The correlation analysis between sequencing depth and mitochondrial genes using Pearson’s method, NA represents no correlation; **B** The correlation analysis between the number of genes and sequencing depth using Pearson’s method, the Pearson correlation coefficient was 0.89; **C** The sequencing depth of 9885 cells from 6 ovarian cancer patients; **D** The number of genes of 9885 cells from 6 ovarian cancer patients; **E** Detection of the highly variable genes across the cells in volcano plot, the top 10 genes were marked out; **F** PCA plot of scRNA-seq samples from 6 patients; **G** The *p* values of PCs from 1–20 calculated by JackStraw function; **H** The standard deviation of 1–30 PCs calculated using ElbowPlot function; **I** Calculation of the cumulative percentages for each PC, 18 is the last point where change of % of variation is more than 0.1%; **H** The t-SNE algorithm divided the cells into 18 clusters by 18 PCs; **I** 18 cell clusters were annotated into 14 cell types

A total of 9885 single-cell samples were used for downstream analysis. The correlation analysis between sequencing depth and mitochondrial genes using Pearson’s method is shown in Fig. 2 A. NA represents no correlation. The Pearson correlation analysis of sequencing depth and number of genes showed a positive correlation with a coefficient of 0.89 (Fig. 2B). The sequencing depth and number of genes in 9885 cells from 6 patients are shown in Fig. 2 C and D. The highly variable genes across the cells are shown in a volcano plot (Fig. 2E). PCA was carried out based on highly variable genes (Fig. 2 F). The definition of the principal components (PC) value depended on 3 approaches: Dimheatmap (Supplementary Fig. 1), the JackStraw function was used to resample the test and calculate the *P*-values of 1–20 PCs (Fig. 2G), and the ElbowPlot function (Fig. 2 H) was used based on the standard deviation. Since there was no obvious elbow point and the *p* values were all < 0.01, we calculated cumulative percentages for each PC. The result shown in Fig. 2I demonstrates that 18 is the last point where the change in % of variation is more than 0.1%. Therefore, we ultimately choose 18 as the PC value. The t-SNE algorithm was used for nonlinear dimension reduction and successfully clustered the single-cell samples into 18 clusters (Fig. 2 J). A total of 2492 genes were used as marker genes for 18 cell clusters, and the top 10 significantly differentially expressed marker genes of each cluster are shown in a heatmap (Supplementary Fig. 2). Afterwards, the 18 cell clusters were annotated into 14 cell types (Fig. 2 K).

### Reconstruction of differentiation trajectories of ovarian cancer omentum metastasis sites

**Fig. 3:**
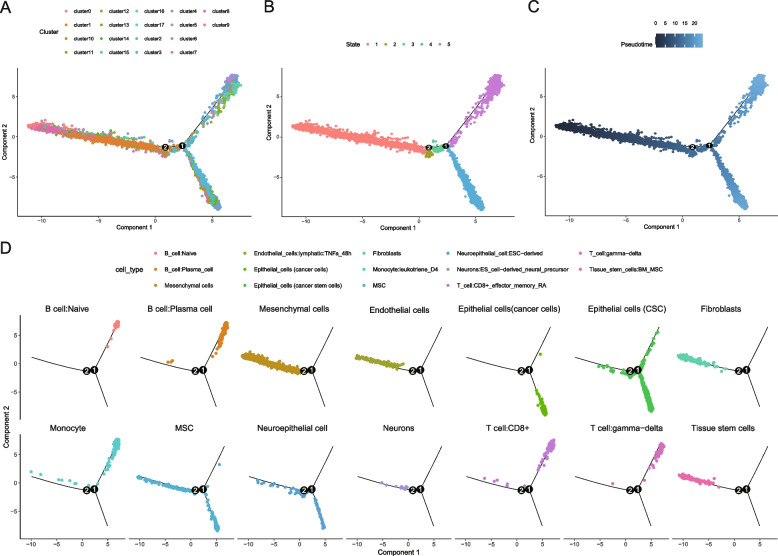
Reconstruction of differentiation trajectories of ovarian cancer omentum metastasis sites. **A** The trajectory plot of 18 clusters using monocle analysis; **B** The trajectory plot of 5 cell states; **C** The trajectory plot in pseudotime, the darker the color is, the default starting point is represented, and the lighter the color is, the farther it is from the starting point of the pseudotimeline; **D** The trajectory plot of 14 cell types in 5 states

The differentiation trajectories of ovarian cancer omentum metastasis were reconstructed with the monocle package and ordered based on 2492 marker genes of 18 cell clusters calculated by Seurat before. The cell clusters, state estimation and pseudotime analysis of single cells are shown in Fig. 3 A, B and C. The distribution of various cell types in different states is shown in Fig. 3D. Except for epithelial cells (cancer cells) and naive B cells, almost every type of cell appeared in state 1; the cell types in state 2 were mainly epithelial cells (CSCs, cancer stem cells), MSCs, and neuroepithelial cells; the cell composition of state 3 was similar to that of state 2, but a few fractions of monocytes and T cells: CD8+; the cell types in state 4 included epithelial cells (cancer cells), epithelial cells (CSCs), MSCs and neuroepithelial cells; while in state 5, the cell types were naive B cells, plasma cells (B cells), epithelial cells (cancer cells), epithelial cells (CSCs), monocytes, MSCs, CD8 + T cells and gamma-delta T cells. From state 1 to state 5, there were 316, 225, 98, 283, and 464 characteristic genes, respectively (Supplementary Files 1, 2, 3, 4 and 5). After merging and removing duplicated genes, 781 characteristic genes of cell differentiation trajectories in the omentum metastasis site were obtained altogether.

### The relevance between cell state characteristic genes and the clinical features of ovarian cancer

**Fig. 4:**
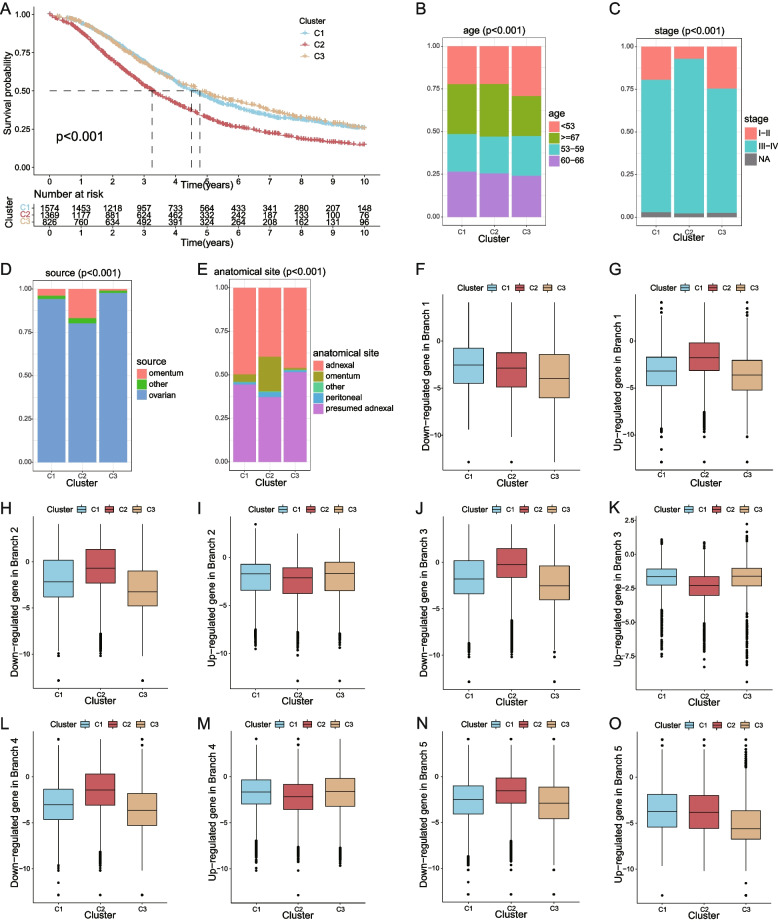
The relevance between cell state characteristic genes and the clinical features of ovarian cancer. **A** K-M survival curve of 3 clusters; **B** Age distribution in each cluster; **C** Proportions of tumor stages in each cluster; **D** Distribution of sample sources in each cluster; **E** Distribution of anatomical sites in each cluster; **F-O** Expression of upregulated and downregulated characteristic genes in 5 states of patients in 3 clusters

Among the 513 genes detected by GSE132342, 74 genes belong to the 781 characteristic genes of cell differentiation trajectories in the omentum metastasis site. With a K value determined as 3 (Supplementary Fig. 3), the 3769 patients in GSE132342 were distributed into 3 clusters according to the expression level of those 74 genes. The K-M survival curve indicated a significantly worse prognosis in patients in cluster 2 (Fig. 2). 4 A), with *P* < 0.01. Figure 4B shows that patients in cluster 3 had a significantly younger age structure (*P* < 0.01), which might be the reason for the best prognosis of this cluster. Cluster 2 had a significantly higher proportion of advanced patients (*P* < 0.01) (Fig. 3 C), and it is widely known that the prognosis of patients in the later period is poor. In addition, the scale of the omentum in both the sample and anatomical site in cluster 2 was significantly larger than that in the other two clusters (Fig. 3D, E). This suggested that patients with omental metastasis were associated with poor survival. The expression levels of characteristic genes in five cell states of patients in three clusters were analyzed to find the possible relations of cell states and the cells in the samples of the patients in three clusters (Fig. 4 F-O).

### Analysis of the TME, immune cell infiltration and immune checkpoints

**Fig. 5:**
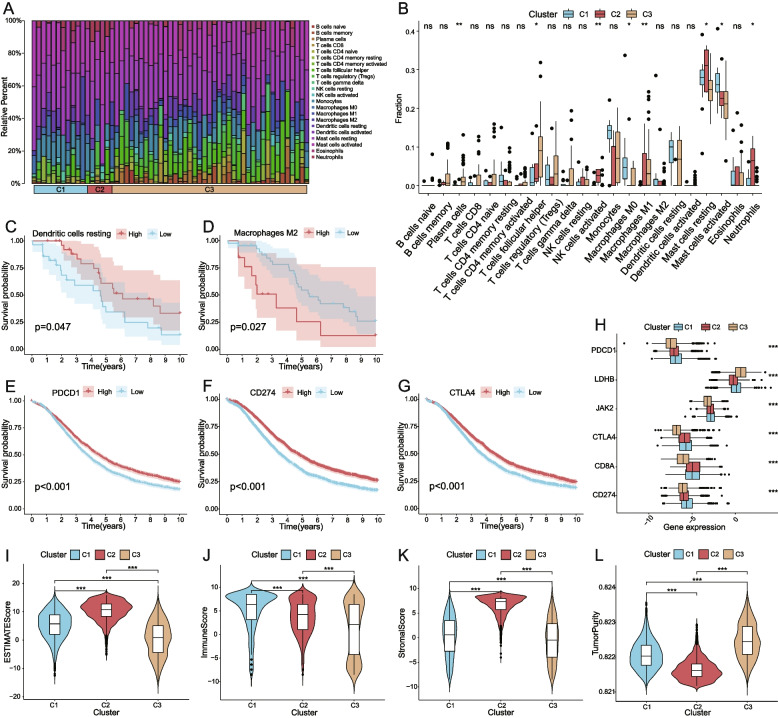
Analysis of the TME, immune cell infiltration and immune checkpoints. **A** The proportion of 22 immune cells built on 3 clusters; **B** The difference of the fraction of 22 immune cells in 3 clusters; **C** The relationship between the proportion of dendritic cells resting and survival; **D** The relationship between the proportion of macrophages M2 cells and survival; **E-G** The relationship between the expression level of PDCD1, CD274, CTLA4 and survival; **H** The expression of 6 immune checkpoint genes with significant differences among the 3 clusters; **I-L** The ESTIMATE Score, Immune Score, Stromal Score, Tumor Purity were significantly different between 3 clusters. (*p < 0.05, **p < 0.01, ***p < 0.001, ns: nonsignificance)

The proportion of 22 immune cells in 3 clusters was evaluated by CIBERSORT, and only samples with a *P-*value < 0.05 were selected for comparison (Fig. 5 A). The proportion of 8 of the 22 immune cells showed significant differences among the 3 clusters (Fig. 5B). Samples in cluster 1 had a higher ratio of activated mast cells and macrophage M0; cluster 2 had a higher ratio of activated NK cells, activated dendritic cells and neutrophils; and cluster 3 had a higher ratio of plasma cells, follicular helper T cells, and M1 macrophages. Through K-M analysis, it was found that among the patients with GSE132342, the higher dendritic cell resting proportion and the lower macrophage M2 proportion had a more favorable prognosis (Fig. 5 C-D). Currently, the main targets of immunotherapy are PD-1, PD-L and CTLA4; PD-1 is encoded by PDCD1, and PD-L1 is encoded by CD274. K-M survival analysis of CD274, CTLA4, PDCD1 and survival showed that high expression levels of CD274, CTLA4 and PDCD1 were associated with better prognosis (Fig. 5E-G). However, the expression level of cluster 2 with the worst survival was not the lowest among the 3 clusters (Fig. 5H). The TME analysis displayed the highest ESTIMATE score and stromal score in cluster 2, the highest immune score in cluster 1 and the highest tumor purity in cluster 3 (Fig. 5I-L).

### Construction of a nomogram model based on the OMAG risk signature and clinicopathological characteristics

**Fig. 6:**
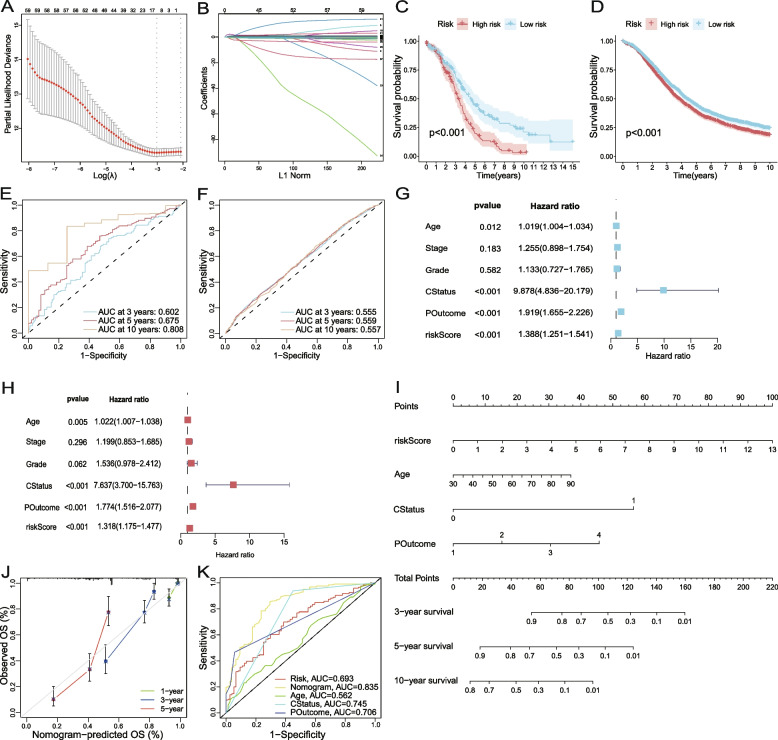
Construction of a nomogram model based on the OMAG risk signature and clinicopathological characteristics. **A** The confidence interval under each lambda; **B** The trajectory of each independent variable: the horizontal axis represents the log value of the independent variable lambda, and the vertical axis represents the coefficient of the independent variable. **C** Survival difference in high- and low-risk scores of the training set (TCGA-OV); **D** Survival difference in high- and low-risk scores of the test set (GSE132342); **E** The prognostic value of the 6-OMAGs signature was evaluated using the ROC curves in the training set (TCGA-OV); **F** The prognostic value of the 6-OMAGs signature was evaluated using the ROC curves in the test set (GSE132342). **G** Univariate Cox regression analyses of the 6-OMAGs and clinicopathological data; **H** Multivariate Cox regression analysis of the 6-OMAGs and clinicopathological data; **I** The nomogram model was constructed to predict the 3-, 5-, and 10-year survival of ovarian cancer patients. CStatus for person neoplasm cancer status, 0 for tumor free, 1 for with tumor; POutcome for primary therapy outcome success, 1 for complete remission/response, 2 for partial remission/response, 3 for stable disease, 4 for progressive disease. **J** The calibration curve of the nomogram at 1, 3, and 5 years; **K** The ROC curve of the nomogram at 5 years

The 74 cell state characteristic genes used to cluster patients in GSE132342 were passed for the candidate genes of OMAGs. The expression profiles of these 74 genes were extracted from bulk RNA-seq data of TCGA-OV, and the candidates were reduced to 10 genes by using LASSO regression (Fig. 6 A-B). Multivariate Cox regression was then used to determine the final 6-OMAGs and their correlation coefficients (Table 1). The formula was Riskscore = 0.307*TIMP3 + 3.516*FBN1–0.109*IGKC + 0.365*RARRES1 + 0.209*RPL21 + 0.870*UCHL1. Taking TCGA-OV as the training set and GSE132342 as the test set, risk scores were calculated in patients of both datasets, and the K-M survival curves reflected a better prognosis of patients with low risk scores (Fig. 6 C-D). The sensitivity in both sets was assessed by receiver operating characteristic (ROC) curves. The area under the curve (AUC) of TCGA-OV was 0.602 at 3 years, 0.675 at 5 years and 0.808 at 10 years (Fig. 6E), which increased with time, while GSE132342 was stable above 0.55 (Fig. 6 F). Univariate Cox regression and multivariate Cox regression were used to analyze the correlation between clinicopathological characteristics and survival of patients in TCGA-OV successively. In univariate regression analysis, the *P*-values of stage and grade were > 0.1, and in multivariate Cox regression, the *P*-values were also > 0.05 (Fig. 6G-H). However, it is well known that stage and grade have important value in prognosis, and the possible reason for this result might be that most patients in TCGA-OV were stage III-IV and grade 3. Finally, a nomogram related to the prognosis of ovarian cancer according to risk scores and clinicopathological characteristics was constructed (Fig. 6I). The ROC curves showed that the accuracy of this nomogram at 5 years was 0.835 (Fig. 6 K).

**Table 1:** The correlation coefficient and *P*-value of the 6-OMAGs

ID	coef	HR	HR.95 L	HR.95 H	*P-*value
TIMP3	0.306572630383264	1.35876015116403	1.10849467977926	1.66552819970137	0.00316056503098236
FBN1	3.51582487967028	33.6436684591936	1.95070915522894	580.248687692912	0.0155261896254875
IGKC	-0.10885566235626	0.896859858795168	0.83457415677356	0.963794049683628	0.00303526487570912
RARRES1	0.364589124350441	1.43992225759669	1.09997372335329	1.88493239784095	0.00796736160943154
RPL21	0.209439598882362	1.23298689907489	1.02201637531489	1.48750717699794	0.0287125482536819
UCHL1	0.87010129427505	2.38715264617469	0.936784113156544	6.08304269479703	0.0682829566218594

### Analysis of the expression and function of 6-OMAGs

**Fig. 7:**
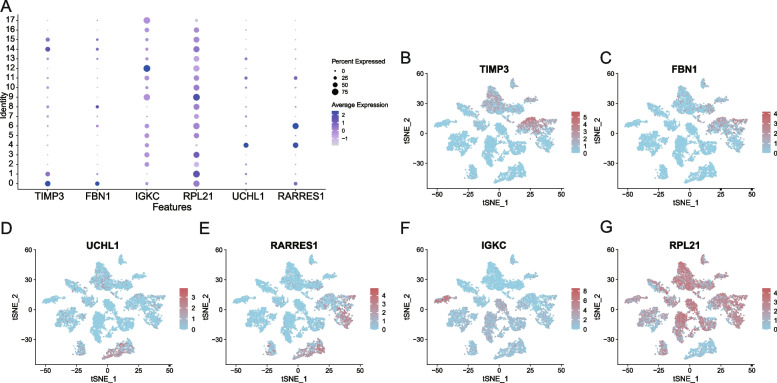
Analysis of the expression and function of 6-OMAGs. **A** Bubble plot of the 6-OMAGs expression level in 18 cell clusters; **B-G** tSNE maps of the expression of 6-OMAGs in 18 cell clusters

The expression levels of 6-OMAGs in 18 cell clusters of ovarian cancer omentum metastasis sites were analyzed (Fig. 7 A). TIMP3 had a higher expression in clusters 0, 1, 14, and 15, which corresponded to mesenchymal cells, endothelial cells and tissue stem cells (Fig. 7B); FBN1 had a higher expression in clusters 0 and 8, which corresponded to mesenchymal cells (Fig. 7 C); UCHL1 had a higher expression in cluster 4, which corresponded to epithelial cells (CSCs) (Fig. 7D); RARRES1 had a higher expression in clusters 4 and 6, which corresponded to epithelial cells (CSCs) and fibroblasts (Fig. 7E); IGKC was expressed in multiple clusters but had a significantly higher average expression in cluster 12, which corresponded to B cells: plasma (Fig. 7 F); RPL21 was expressed in almost all clusters, except cluster 4, which corresponded to epithelial cells (CSCs) (Fig. 7G).

The correlation between the expression of 6-OMAGs and 20 tumor-related pathway scores (tumor inflammation signature, cellular response to hypoxia, tumor proliferation signature, EMT markers, ECM-related genes, angiogenesis, apoptosis, DNA repair, G2/M checkpoint, inflammatory response, PI3K/AKT/mTOR pathway, P53 pathway, MYC targets, TGFβ, IL-10 anti-inflammatory signaling pathway, genes upregulated by reactive oxygen species (ROS), DNA replication, collagen formation, degradation of ECM, ferroptosis, generated by ssGSEA (Supplementary File 6)) were analyzed by Spearman correlation (Supplementary Figs. 4, 5, 6, 7, 8 and 9). *P*-value < 0.05 and ρSpearman > 0.3 are listed below (Table 2). The expression of FBN1, RARRES1, and TIMP3 was positively correlated with multiple tumor-related pathways.

**Table 2:** The ρSpearman value and *P*-value of the 6-OMAGs correlated with pathways

Pathway	Gene	ρSpearman	*P*-value
**Tumor Inflammation Signature**	RARRES1	0.37	1.53e − 13
**EMT markers**	FBN1	0.75	3.62e − 69
	RARRES1	0.32	3.84e − 10
	TIMP3	0.60	1.39e − 38
**ECM-related genes**	FBN1	0.70	8.72e − 56
	RARRES1	0.35	4.24e − 12
	TIMP3	0.59	2.55e − 36
**Angiogenesis**	FBN1	0.76	1.02e − 71
	RARRES1	0.40	8.76e − 16
	TIMP3	0.59	5.05e − 36
**Apoptosis**	FBN1	0.54	2.48e − 29
	RARRES1	0.42	2.67e − 17
	TIMP3	0.35	4.28e − 12
**Inflammatory response**	FBN1	0.46	4.35e − 21
	RARRES1	0.47	1.37e − 21
**PI3K-AKT-mTOR pathway**	FBN1	0.35	2.61e − 12
	RARRES1	0.33	6.89e − 11
**P53 pathway**	FBN1	0.51	7.82e − 27
	RARRES1	0.39	5.46e − 15
	TIMP3	0.37	1.27e − 13
**TGFβ**	FBN1	0.75	1.59e − 69
	TIMP3	0.67	5.73e − 50
**IL-10 Anti-inflammatory Signaling Pathway**	FBN1	0.32	2.36e − 10
	RARRES1	0.43	1.21e − 18
**Genes up-regulated by reactive oxygen species (ROS)**	RARRES1	0.45	2.85e − 20
**Collagen formation**	FBN1	0.84	2.12e − 101
	TIMP3	0.75	7.58e − 68
**Degradation of ECM**	FBN1	0.83	1.35e − 96
	TIMP3	0.70	1.45e − 59
**Ferroptosis**	FBN1	0.47	4.32e − 22
	RARRES1	0.41	1.95e − 16

**Fig. 8:**
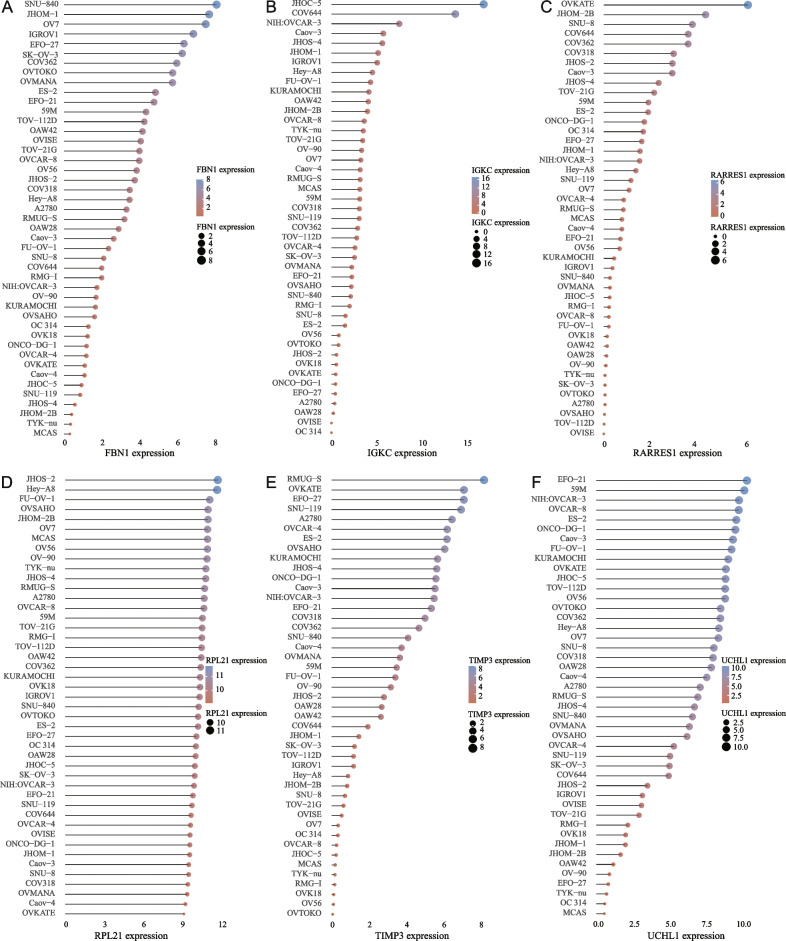
The expression distribution of 6-OMAGs mRNA in 45 human ovarian cancer cell lines. The x-axis represents the expression distribution of mRNA, the y-axis represents different cell lines, different colors and the size of dots represent expression

The expression distribution of 6-OMAGs mRNA in 45 human ovarian cancer cell lines obtained from the CCLE dataset demonstrated that there were large variations in the expression levels of FBN1, TIMP3, and UCHL1 between cell lines (Fig. 8 A, E, F). The expression levels of IGKC and RARRES1 were overall low in 45 cell lines and only higher than 5 in specific cell lines (Fig. 8B, C). The expression level of RPL21 in all cell lines was relatively average and high (Fig. 8D).

## Discussion

In this study, the scRNA-seq data of omentum metastasis sites from 6 ovarian cancer patients, GEO microarray data of 3769 patients, TCGA-OV bulk RNA-seq data of 379 patients and clinical information were combined to construct a prognostic prediction model of ovarian cancer composed of a 6-OMAGs signature and 3 clinicopathological features.

In comparison with the original authors [12], they performed the hierarchical clustering with a resolution of 0.2, PCs varied from 10 to 20 depending on the sample and UMAP dimension reduction, with 12 clusters detected. While we selected a PC value of 18, a resolution of 0.5, and t-SNE dimensionality reduction analysis identified the single cells into 18 clusters. The difference between these parameters and methods will change the cluster distribution and mapping. The original authors adopted a sophisticated way of cell annotation, integrating canonical genes, functional categories, and cell line correlation. Finally, they annotated the 12 clusters into nine types of cells and three unidentified clusters. We mainly used the SingleR package and adjusted the annotation results according to the marker genes in the literature, and obtained 14 cell types. In our cell annotation results, six cell types are the same as those of the original author, including epithelial cells, fibroblasts, mesenchymal stem cells, endothelial cells, B cells, and plasma B cells. But there are also some differences. For example, the original authors only annotated T cells, but we divided T cells into two different clusters, they annotated macrophages, but we only had monocyte. Interestingly, in our results, the epithelial cells (cancer cells) were separated into three clusters. This may be attributed to the heterogeneity of tumors. It may be valuable to further investigate the differences between these epithelial cell clusters in our follow-up research, which may provide some ideas for the precise treatment of ovarian cancer.

The differentiation trajectory analysis of ovarian cancer omentum metastasis sites revealed that each cell type was not necessarily in only one state, and each state contained multiple cell types. Cells might express diverse sets of genes during different states; when cells move between states, some genes might be silenced, while some might be newly activated to carry out their work. Hence, these 781 characteristic genes might have a connection with the composition, function and state of cells in the TME and are likely related to the progression of ovarian cancer.

When the patients from GSE132342 were grouped into 3 clusters in accordance with the expression of 74 cell state marker genes, patients in cluster 1 had relatively low expression in the downregulated genes of states 2, 3, 4, and 5 and relatively high expression in the upregulated genes, and it was speculated that the samples’ TME might be in states 2, 3, 4, and 5. Similarly, it could be inferred that the TME of samples in cluster 2 might be in state 1, and the TME of samples in cluster 3 might be in states 2, 3, and 4. Comparing this conjecture with the conclusion of the original author of scRNA-seq data, they found high T cell infiltration group had an anti-tumor response. Our results showed that T cells were mainly concentrated in state 5, while patients with cluster 1 had similar expression characteristics to those with state 5, and the prognosis of patients with cluster 1 was significantly better than that of patients with cluster 2, which was also consistent with the original author’s conclusion.

The clustering results according to the marker genes of cell states were supplemented and examined by CIBERSORT and TME analysis. This was helpful to further analyze the relationship between the omental metastasis sites’ characteristic genes of cell state and ovarian cancer prognosis. Cluster 2 had a higher percentage of neutrophils, which proved to be one of the cells facilitating the formation of omentum PMNs [4]. Among the immune cells with a higher proportion in cluster 3, follicular helper T cells [32] and macrophage M1 [33] were recognized to have antitumor effects, similar to plasma cells. The original author of the scRNA-seq data discovered a unique plasmablast and plasma B cell clusters that may contribute to the immune response within the TME [12], which might be the reason why patients in cluster 3 had a better prognosis.

The immune checkpoint analysis displayed seemingly contradictory results between expression level and prognosis; however, research has also shown that high expression of PD1 and PD-L1 is associated with favorable outcome in lung cancer of early-stage but an adverse outcome in late-stage [34]. The ratio of stage I-II patients in cluster 3 was much higher than that in cluster 2, and it might be the dual effect of PD1 and PD-L1 on prognosis that leads to this result.

The TME analysis also suggested that there might be a correlation between the cell states and the sample clustering. As presumed previously, the cells of cluster 1 samples were mainly in states 2, 3, 4, and 5; cluster 2 was mainly in state 1; and cluster 3 was mainly in states 2, 3, and 4. The expression characteristics of state 5 were unique to cluster 1, and state 5 contained a large quantity of immune cells. The expression characteristics of state 1 were unique to cluster 3, which consisted of many stromal cells. In comparison, cluster 3 indeed had the highest tumor cell content. This result implied that the progression and prognosis of tumors not only depended on the characteristics of tumor cells but also relied on interactions within the niche.

The 6-OMAGs finally screened were TIMP3, FBN1, IGKC, RARRES1, RPL21 and UCHL1. TIMP3 (tissue inhibitors of metalloproteinase 3) was shown to be associated with metastasis and poor survival in serous ovarian cancer by regulating TGF-beta signaling [35]. Our results also found that TIMP3 was positively correlated with TGF beta; in addition, TIMP3 was positively related to EMT, extracellular matrix (ECM), angiogenesis, apoptosis, the P53 pathway, ECM degradation, and collagen formation, which are the potential mechanisms of its tumor-promoting effect. TIMP3 had a diverse expression level among 45 human ovarian cancer cell lines, which might be due to their different characteristics. In single-cell samples, TIMP3 had a significantly high expression in mesenchymal cells, and studies demonstrated that TIMP3 can promote tumor progression through EMT [36, 37], which also verifies our conclusion.

FBN1 (fibrillin-1) has been reported to enhance the cisplatin resistance of ovarian cancer by being involved in angiogenesis and glycolysis [38]. Our study also found a strong correlation between the expression of FBN1 and angiogenesis with a ρSpearman of 0.76. Our results inferred that it is related to EMT with a ρSpearman of 0.75, which might be the reason why it is highly expressed in cells annotated as mesenchymal. In addition, we also found a considerable correlation of its expression with ECM, apoptosis, inflammatory response, PI3K/AKT/mTOR pathway, P53 pathway, TGFβ, IL-10 anti-inflammatory signaling pathway, collagen formation and ferroptosis. It also had a diverse expression level in different cell lines, and its complex mechanism in cancer progression remains to be further elucidated.

IGKC (immunoglobulin kappa C), expressed in plasma cells, has been identified as one of the top genes of a prognostic B cell metagene in breast cancer, correlated with favorable prognosis and response to chemotherapy [39]. A study showed that plasma cell infiltration in epithelial ovarian cancer has a significant impact on tumor progression and prognosis [40], and high expression of IGKC is associated with good outcome [41]. In our study, although it was widely expressed in a variety of cell types, it was only significantly overexpressed in B cells: plasma cells. Our pathway correlation analysis also did not find a significant positive correlation between IGKC and 20 tumor-related pathways, and its expression level in ovarian cancer cell lines was generally low, indicating that its high expression in ovarian cancer may be detrimental to the proliferation of ovarian cancer cells.

Ribosomal proteins (RPs) are involved in the cellular process of translation, and in recent years, RP dysfunction has been found in tumors, such as mutation, expression level changes and correlation with differentiation [42–44]. RPL21 (ribosomal protein gene 21) has been found to be associated with the proliferation of pancreatic cancer cells [45] and may be used as a potential marker for cervical intraepithelial neoplasia [46], but there are few reports in ovarian cancer. As a cellular translation process-related gene, it is not surprising that RPL21 is widely and highly expressed in various cell clusters and 45 ovarian cancer cell lines; however, our study revealed significantly low expression in the cluster of epithelial cells (CSCs), suggesting that the change in its expression may be related to the increased stemness of ovarian cancer cells.

UCHL1 (ubiquitin carboxyl-terminal esterase L1) is an oncogene encoding a deubiquitinating enzyme that adjusts the balance between mTOR complexes [47] and plays a significant role in the ubiquitin system as well as different cellular processes [48]. It has been widely studied and confirmed to be related to tumor progression, such as prostate cancer, lymphoma, lung cancer, breast cancer, neuroblastoma, etc. [48–51]. In ovarian cancer, UCHL1 may be one of the markers related to early stages of high-grade serous carcinoma, immunogenicity [52, 53], and cisplatin resistance [54, 55]. Our results showed that it was specifically overexpressed in epithelial cells (CSCs), while the expression levels varied significantly in different ovarian cancer cell lines. Interestingly, no significant correlation was found between UCHL1 and tumor-related functions. This suggests that UCHL1 is related to the stemness of ovarian cancer cells, but its specific mechanism requires further investigation.

RARRES1 (retinoic acid receptor responder 1) is one of the common methylated loci in several cancers and is believed to be a putative tumor suppressor gene [56, 57]. Promoter hypermethylation and loss of RARRES1 seem to facilitate cancer progression [58]. In this study, RARRES1 was found to be highly expressed in epithelial cells (CSCs) and fibroblasts, and studies have revealed the possibility that RARRES1 can be used as a carcinoma-associated fibroblast (CAF) marker gene in breast cancer and may lead to chemotherapy resistance [59, 60]. It could be speculated that fibroblast cells with high RARRES1 expression might be transformed into CAFs after remodeling by tumor cells, making the omental microenvironment easier for tumor cell migration/invasion. Compared with the other five OMAGs, the overall expression level of RARRES1 in ovarian cancer cell lines was not high. Moreover, although it has a significant correlation in multiple cancer-related pathways, the correlation coefficients are between 0.3 and 0.5, which does not show a strong correlation. This may be related to the stemness of tumor cells, which still needs further research.

Although there are various models related to the prognosis of ovarian cancer, few focus on omentum metastasis. Our study has proven to a certain extent that there is a correlation between the cell states of omental metastasis sites and the prognosis of ovarian cancer. For ovarian cancer patients who have not yet metastasized, the marker genes of cell states also have prognostic value. This may be because the omentum microenvironment with these characteristics is more likely to form PMN during the interaction with residual tumor cells, making it for proliferation and colonization. There are still many deficiencies and limitations in our research. Although GSE132342 contains considerable samples for analysis, one of its nonnegligible shortcomings is that it only has the expression information of 513 genes, and many valuable characteristic genes of cell states are excluded. The number of state marker genes decreased from 781 to 74. On the other hand, these 513 genes are also supported by high-level evidence that they are related to the prognosis of ovarian cancer. When we intersected these 513 genes with the characteristic genes of cell states, the 74 genes obtained were more likely to be related to both the state of ovarian cancer omental tumor cells and the prognosis of ovarian cancer. Apart from this, the study lacks first-hand data, and the potential mechanism has not been verified by experiments, so more clinical trials and laboratory experiments are needed in follow-up research.

## Supplementary Information

**Additional file 1.**

**Additional file 2.**

**Additional file 3.**

**Additional file 4.**

**Additional file 5.**

**Additional file 6.**

**Additional file 7.**

**Additional file 8.**

**Additional file 9.**

**Additional file 10.**

**Additional file 11.**

**Additional file 12.**

**Additional file 13.**

**Additional file 14.**

**Additional file 15.**

## Data Availability

Please contact the corresponding author Wenping Lu (lu_wenping@sina.com).

## References

[1] Menon U, Gentry-Maharaj A, Burnell M (2021). Ovarian cancer population screening and mortality after long-term follow-up in the UK Collaborative Trial of Ovarian Cancer Screening (UKCTOCS): a randomised controlled trial. Lancet.

[2] Torre LA, Trabert B, DeSantis CE (2018). Ovarian cancer statistics, 2018. CA Cancer J Clin.

[3] Pradeep S, Kim SW, Wu SY (2014). Hematogenous metastasis of ovarian cancer: rethinking mode of spread. Cancer Cell.

[4] Lee W, Ko SY, Mohamed MS (2019). Neutrophils facilitate ovarian cancer premetastatic niche formation in the omentum. J Exp Med.

[5] Bookman MA, Okamoto A, Stuart G (2017). Harmonising clinical trials within the Gynecologic Cancer InterGroup: consensus and unmet needs from the Fifth Ovarian Cancer Consensus Conference. Ann Oncol.

[6] Girolimetti G, De Iaco P, Procaccini M (2017). Mitochondrial DNA sequencing demonstrates clonality of peritoneal implants of borderline ovarian tumors. Mol Cancer.

[7] Curtis M, Kenny HA, Ashcroft B (2019). Fibroblasts Mobilize Tumor Cell Glycogen to Promote Proliferation and Metastasis. Cell Metab.

[8] Etzerodt A, Moulin M, Doktor TK (2020). Tissue-resident macrophages in omentum promote metastatic spread of ovarian cancer. J Exp Med.

[9] Zhang AW, McPherson A, Milne K (2018). Interfaces of Malignant and Immunologic Clonal Dynamics in Ovarian Cancer. Cell.

[10] Liu Q, Zhang H, Jiang X (2017). Factors involved in cancer metastasis: a better understanding to “seed and soil” hypothesis. Mol Cancer.

[11] Potter SS (2018). Single-cell RNA sequencing for the study of development, physiology and disease. Nat Rev Nephrol.

[12] Olalekan S, Xie B, Back R (2021). Characterizing the tumor microenvironment of metastatic ovarian cancer by single-cell transcriptomics. Cell Rep.

[13] Liu C, Zhang Y, Li X, Wang D (2022). Ovarian cancer-specific dysregulated genes with prognostic significance: scRNA-Seq with bulk RNA-Seq data and experimental validation. Ann N Y Acad Sci.

[14] Millstein J, Budden T, Goode EL (2020). Prognostic gene expression signature for high-grade serous ovarian cancer. Ann Oncol.

[15] Hao Y, Hao S, Andersen-Nissen E (2021). Integrated analysis of multimodal single-cell data. Cell.

[16] Aran D, Looney AP, Liu L (2019). Reference-based analysis of lung single-cell sequencing reveals a transitional profibrotic macrophage. Nat Immunol.

[17] Li L, Dong J, Yan L (2017). Single-Cell RNA-Seq Analysis Maps Development of Human Germline Cells and Gonadal Niche Interactions. Cell Stem Cell.

[18] Winterhoff BJ, Maile M, Mitra AK (2017). Single cell sequencing reveals heterogeneity within ovarian cancer epithelium and cancer associated stromal cells. Gynecol Oncol.

[19] Schelker M, Feau S, Du J (2017). Estimation of immune cell content in tumour tissue using single-cell RNA-seq data. Nat Commun.

[20] Neradil J, Veselska R (2015). Nestin as a marker of cancer stem cells. Cancer Sci.

[21] Qiu X, Mao Q, Tang Y (2017). Reversed graph embedding resolves complex single-cell trajectories. Nat Methods.

[22] Ritchie ME, Phipson B, Wu D (2015). limma powers differential expression analyses for RNA-sequencing and microarray studies. Nucleic Acids Res.

[23] Wilkerson MD, Hayes DN (2010). ConsensusClusterPlus: a class discovery tool with confidence assessments and item tracking. Bioinformatics.

[24] Robertson AG, Shih J, Yau C (2017). Integrative Analysis Identifies Four Molecular and Clinical Subsets in Uveal Melanoma. Cancer Cell.

[25] Yoshihara K, Shahmoradgoli M, Martinez E (2013). Inferring tumour purity and stromal and immune cell admixture from expression data. Nat Commun.

[26] Newman AM, Steen CB, Liu CL (2019). Determining cell type abundance and expression from bulk tissues with digital cytometry. Nat Biotechnol.

[27] Tibshirani R, Bien J, Friedman J (2012). Strong rules for discarding predictors in lasso-type problems. J R Stat Soc Series B Stat Methodol.

[28] Balachandran VP, Gonen M, Smith JJ, DeMatteo RP (2015). Nomograms in oncology: more than meets the eye. Lancet Oncol.

[29] Hanzelmann S, Castelo R, Guinney J (2013). GSVA: gene set variation analysis for microarray and RNA-seq data. BMC Bioinformatics.

[30] Wei J, Huang K, Chen Z (2020). Characterization of Glycolysis-Associated Molecules in the Tumor Microenvironment Revealed by Pan-Cancer Tissues and Lung Cancer Single Cell Data. Cancers (Basel).

[31] Ghandi M, Huang FW, Jane-Valbuena J (2019). Next-generation characterization of the Cancer Cell Line Encyclopedia. Nature.

[32] Cui C, Wang J, Fagerberg E (2021). Neoantigen-driven B cell and CD4 T follicular helper cell collaboration promotes anti-tumor CD8 T cell responses. Cell.

[33] Locati M, Curtale G, Mantovani A (2020). Diversity, Mechanisms, and Significance of Macrophage Plasticity. Annu Rev Pathol.

[34] Chang CH, Shih AC, Chang YH (2021). The Prognostic Significance of PD1 and PDL1 Gene Expression in Lung Cancer: A Meta-Analysis. Front Oncol.

[35] Cheon DJ, Tong Y, Sim MS (2014). A collagen-remodeling gene signature regulated by TGF-beta signaling is associated with metastasis and poor survival in serous ovarian cancer. Clin Cancer Res.

[36] Su CW, Chang YC, Chien MH (2019). Loss of TIMP3 by promoter methylation of Sp1 binding site promotes oral cancer metastasis. Cell Death Dis.

[37] Li W, Song YY, Rao T (2022). CircCSNK1G3 up-regulates miR-181b to promote growth and metastasis via TIMP3-mediated epithelial to mesenchymal transitions in renal cell carcinoma. J Cell Mol Med.

[38] Wang Z, Chen W, Zuo L (2022). The Fibrillin-1/VEGFR2/STAT2 signaling axis promotes chemoresistance via modulating glycolysis and angiogenesis in ovarian cancer organoids and cells. Cancer Commun (Lond).

[39] Whiteside TL, Ferrone S (2012). For breast cancer prognosis, immunoglobulin kappa chain surfaces to the top. Clin Cancer Res.

[40] Lundgren S, Berntsson J, Nodin B (2016). Prognostic impact of tumour-associated B cells and plasma cells in epithelial ovarian cancer. J Ovarian Res.

[41] Schmidt M, Hellwig B, Hammad S (2012). A comprehensive analysis of human gene expression profiles identifies stromal immunoglobulin kappa C as a compatible prognostic marker in human solid tumors. Clin Cancer Res.

[42] Warner JR, McIntosh KB (2009). How common are extraribosomal functions of ribosomal proteins?. Mol Cell.

[43] Loreni F, Mancino M, Biffo S (2014). Translation factors and ribosomal proteins control tumor onset and progression. how? Oncogene.

[44] Doherty L, Sheen MR, Vlachos A (2010). Ribosomal protein genes RPS10 and RPS26 are commonly mutated in Diamond-Blackfan anemia. Am J Hum Genet.

[45] Li C, Ge M, Chen D (2020). RPL21 siRNA Blocks Proliferation in Pancreatic Cancer Cells by Inhibiting DNA Replication and Inducing G1 Arrest and Apoptosis. Front Oncol.

[46] Suman S, Mishra A, Kulshrestha A (2017). A systems approach for the elucidation of crucial genes and network constituents of cervical intraepithelial neoplasia 1 (CIN1). Mol Biosyst.

[47] Bedekovics T, Hussain S, Feldman AL, Galardy PJ (2016). UCH-L1 is induced in germinal center B cells and identifies patients with aggressive germinal center diffuse large B-cell lymphoma. Blood.

[48] Ummanni R, Jost E, Braig M (2011). Ubiquitin carboxyl-terminal hydrolase 1 (UCHL1) is a potential tumour suppressor in prostate cancer and is frequently silenced by promoter methylation. Mol Cancer.

[49] Ding X, Gu Y, Jin M (2020). The deubiquitinating enzyme UCHL1 promotes resistance to pemetrexed in non-small cell lung cancer by upregulating thymidylate synthase. Theranostics.

[50] Liu S, Gonzalez-Prieto R, Zhang M (2020). Deubiquitinase Activity Profiling Identifies UCHL1 as a Candidate Oncoprotein That Promotes TGFbeta-Induced Breast Cancer Metastasis. Clin Cancer Res.

[51] Gu Y, Lv F, Xue M (2018). The deubiquitinating enzyme UCHL1 is a favorable prognostic marker in neuroblastoma as it promotes neuronal differentiation. J Exp Clin Cancer Res.

[52] Gutkin DW, Shurin MR, El Azher MA (2019). Novel protein and immune response markers of human serous tubal intraepithelial carcinoma of the ovary. Cancer Biomark.

[53] Tangri A, Lighty K, Loganathan J (2021). Deubiquitinase UCHL1 Maintains Protein Homeostasis through the PSMA7-APEH-Proteasome Axis in High-grade Serous Ovarian Carcinoma. Mol Cancer Res.

[54] Cucci MA, Grattarola M, Dianzani C (2020). Ailanthone increases oxidative stress in CDDP-resistant ovarian and bladder cancer cells by inhibiting of Nrf2 and YAP expression through a post-translational mechanism. Free Radic Biol Med.

[55] Jin C, Yu W, Lou X (2013). UCHL1 Is a Putative Tumor Suppressor in Ovarian Cancer Cells and Contributes to Cisplatin Resistance. J Cancer.

[56] Sahab ZJ, Hall MD, Me Sung Y (2011). Tumor suppressor RARRES1 interacts with cytoplasmic carboxypeptidase AGBL2 to regulate the alpha-tubulin tyrosination cycle. Cancer Res.

[57] Peterfi L, Banyai D, Yusenko MV (2020). Expression of RARRES1 and AGBL2 and progression of conventional renal cell carcinoma. Br J Cancer.

[58] Huebner H, Strick R, Wachter DL (2017). Hypermethylation and loss of retinoic acid receptor responder 1 expression in human choriocarcinoma. J Exp Clin Cancer Res.

[59] Li Y, Rong G, Kang H (2017). Taxotere-induced elevated expression of IL8 in carcinoma-associated fibroblasts of breast invasive ductal cancer. Oncol Lett.

[60] Rong G, Kang H, Wang Y (2013). Candidate markers that associate with chemotherapy resistance in breast cancer through the study on Taxotere-induced damage to tumor microenvironment and gene expression profiling of carcinoma-associated fibroblasts (CAFs). PLoS ONE.

