# Supplementary material for: Establishment of an ovarian cancer omentum metastasis-related prognostic model by integrated analysis of scRNA-seq and bulk RNA-seq
Source: J Ovarian Res. 2022 Nov 23;15:123. doi: 10.1186/s13048-022-01059-0 (PMC9686070; doi:10.1186/s13048-022-01059-0)
Supplement: Supplementary file 1 — Additional file 1. [file 13048_2022_1059_MOESM1_ESM.pdf]

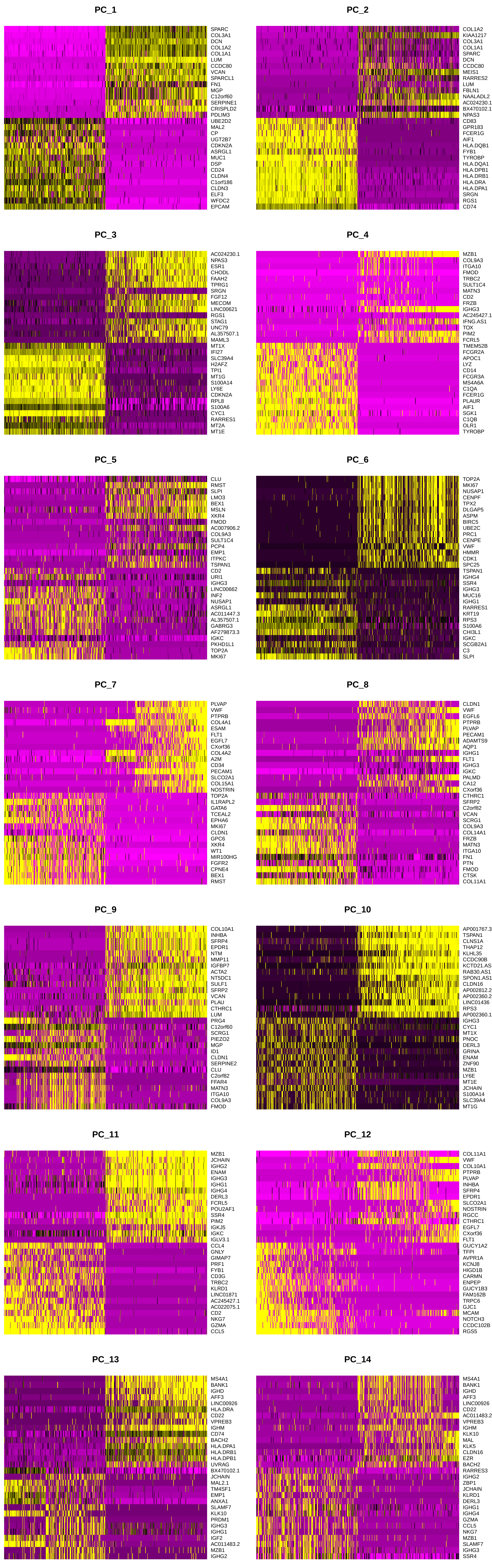
PC_1
SPARC
COL3A1
DCN
COL1A2
COL1A1
LUM
CCDC80
VCAN
SPARCL1
FN1
MGP
C12orf60
SERPINE1
CRISPLD2
PDLIM3
UBE2D2
MAL2
CP
UGT2B7
CDKN2A
ASRGL1
MUC1
DSP
CD24
CLDN4
C1orf186
CLDN3
ELF3
WFDC2
EPCAM
PC_2
COL1A2
KIAA1217
COL3A1
COL1A1
SPARC
DCN
CCDC80
MEIS1
RARRES2
LUM
FBLN1
NAALADL2
AC024230.1
BX470102.1
NPAS3
CD83
GPR183
FCER1G
AIF1
HLA.DQB1
FYB1
TYROBP
HLA.DQA1
HLA.DPB1
HLA.DRB1
HLA.DRA
HLA.DPA1
SRGN
RGS1
CD74
PC_3
AC024230.1
NPAS3
ESR1
CHODL
FAAH2
TPRG1
SRGN
FGF12
MECOM
LINC00621
RGS1
STAG1
UNC79
AL357507.1
MAML3
MT1X
IFI27
SLC39A4
H2AFZ
TPI1
MT1G
S100A14
LY6E
CDKN2A
RPL8
S100A6
CYC1
RARRES1
MT2A
MT1E
PC_4
MZB1
COL9A3
ITGA10
FMOD
TRBC2
SULT1C4
MATN3
CD2
FRZB
IGHG3
AC245427.1
IFNG.AS1
TOX
PIM2
FCRL5
TMEM52B
FCGR2A
APOC1
LYZ
CD14
FCGR3A
MS4A6A
C1QA
FCER1G
PLAUR
AIF1
SGK1
C1QB
OLR1
TYROBP
PC_5
CLU
RMST
SLPI
LMO3
BEX1
MSLN
XKR4
FMOD
AC007906.2
COL9A3
SULT1C4
PCP4
EMP1
ITPKC
TSPAN1
CD2
URI1
IGHG3
LINC00662
INF2
NUSAP1
ASRGL1
AC011447.3
AL357507.1
GABRG3
AF279873.3
IGKC
PKHD1L1
TOP2A
MKI67
PC_6
TOP2A
MKI67
NUSAP1
CENPF
TPX2
DLGAP5
ASPM
BIRC5
UBE2C
PRC1
CENPE
VWF
HMMR
CDK1
SPC25
TSPAN1
IGHG4
SSR4
IGHG3
MUC16
IGHG1
RARRES1
KRT19
RPS3
S100A6
CHI3L1
IGKC
SCGB2A1
C3
SLPI
PC_7
PLVAP
VWF
PTPRB
COL4A1
ESAM
FLT1
EGFL7
CXorf36
COL4A2
A2M
CD34
PECAM1
SLCO2A1
COL15A1
NOSTRIN
TOP2A
IL1RAPL2
GATA6
TCEAL2
EPHA6
MKI67
CLDN1
GPC6
XKR4
WT1
MIR100HG
FGFR2
CPNE4
BEX1
RMST
PC_8
CLDN1
VWF
EGFL6
PTPRB
PLVAP
PECAM1
ADAMTS9
AQP1
IGHG1
FLT1
IGHG3
IGKC
PALMD
CA12
CXorf36
CTHRC1
SFRP2
C2orf82
VCAN
SCRG1
COL9A3
COL14A1
FRZB
MATN3
ITGA10
FN1
PTN
FMOD
CTSK
COL11A1
PC_9
COL10A1
INHBA
SFRP4
EPDR1
NTM
MMP11
IGFBP7
ACTA2
NT5DC1
SULF1
SFRP2
VCAN
PLAU
CTHRC1
LUM
PRG4
C12orf60
SCRG1
PIEZO2
MGP
ID1
CLDN1
SERPINE2
CLU
C2orf82
FFAR4
MATN3
ITGA10
COL9A3
FMOD
PC_10
AP001767.3
TSPAN1
CLNS1A
THAP12
KLHL35
CCDC90B
KCTD21.AS
RAB30.AS1
SPON1.AS1
CLDN16
AP002812.2
AP002360.1
LINC01436
RPS3
AP002360.1
IGHG3
CYC1
MT1X
PNOC
DERL3
GRINA
ENAM
ZNF90
MZB1
LY6E
MT1E
JCHAIN
S100A14
SLC39A4
MT1G
PC_11
MZB1
JCHAIN
IGHG2
ENAM
IGHG3
IGHG1
IGHG4
DERL3
FCRL5
POU2AF1
SSR4
PIM2
IGKJ5
IGKC
IGLV3.1
CCL4
GNLY
GIMAP7
PRF1
FYB1
CD3G
TRBC2
KLRD1
LINC01871
AC245427.1
AC022075.1
CD2
NKG7
GZMA
CCL5
PC_12
COL11A1
VWF
COL10A1
PTPRB
PLVAP
INHBA
SFRP4
EPDR1
SLCO2A1
NOSTRIN
RGCC
CTHRC1
EGFL7
CXorf36
FLT1
GUCY1A2
TFPI
AVPR1A
KCNJ8
HIGD1B
CARMN
ENPEP
GUCY1B3
FAM162B
TRPC6
GJC1
MCAM
NOTCH3
CCDC102B
RGS5
PC_13
MS4A1
BANK1
IGHD
AFF3
LINC00926
HLA.DRA
CD22
VPREB3
IGHM
CD74
BACH2
HLA.DPA1
HLA.DRB1
HLA.DPB1
UVRAG
BX470102.1
JCHAIN
MAL2.1
TM4SF1
EMP1
ANXA1
SLAMF7
KLK10
PRDM1
IGHG3
IGHG1
IGF2
AC011483.2
MZB1
IGHG2
PC_14
MS4A1
BANK1
IGHD
AFF3
LINC00926
CD22
AC011483.2
VPREB3
IGHM
KLK10
MAL
KLK5
CLDN16
EZR
BACH2
RARRES3
IGHG2
ZBP1
JCHAIN
KLRD1
DERL3
IGHG1
IGHG4
GZMA
CCL5
NKG7
MZB1
SLAMF7
IGHG3
SSR4

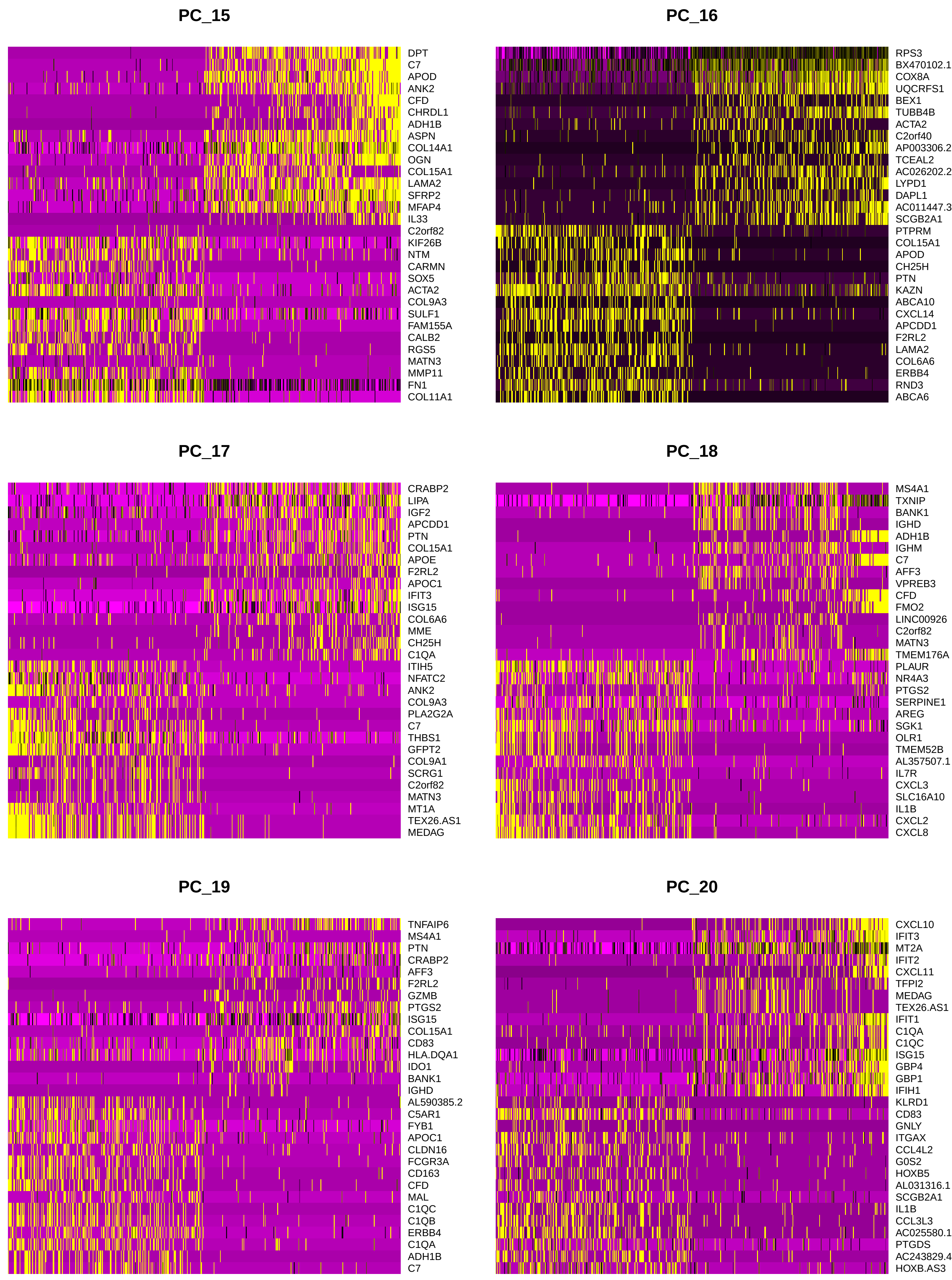
PC_15
DPT
C7
APOD
ANK2
CFD
CHRDL1
ADH1B
ASPN
COL14A1
OGN
COL15A1
LAMA2
SFRP2
MFAP4
IL33
C2orf82
KIF26B
NTM
CARMN
SOX5
ACTA2
COL9A3
SULF1
FAM155A
CALB2
RGS5
MATN3
MMP11
FN1
COL11A1
PC_16
RPS3
BX470102.1
COX8A
UQCRFS1
BEX1
TUBB4B
ACTA2
C2orf40
AP003306.2
TCEAL2
AC026202.2
LYPD1
DAPL1
AC011447.3
SCGB2A1
PTPRM
COL15A1
APOD
CH25H
PTN
KAZN
ABCA10
CXCL14
APCDD1
F2RL2
LAMA2
COL6A6
ERBB4
RND3
ABCA6
PC_17
CRABP2
LIPA
IGF2
APCDD1
PTN
COL15A1
APOE
F2RL2
APOC1
IFIT3
ISG15
COL6A6
MME
CH25H
C1QA
ITIH5
NFATC2
ANK2
COL9A3
PLA2G2A
C7
THBS1
GFPT2
COL9A1
SCRG1
C2orf82
MATN3
MT1A
TEX26.AS1
MEDAG
PC_18
MS4A1
TXNIP
BANK1
IGHD
ADH1B
IGHM
C7
AFF3
VPREB3
CFD
FMO2
LINC00926
C2orf82
MATN3
TMEM176A
PLAUR
NR4A3
PTGS2
SERPINE1
AREG
SGK1
OLR1
TMEM52B
AL357507.1
IL7R
CXCL3
SLC16A10
IL1B
CXCL2
CXCL8
PC_19
TNFAIP6
MS4A1
PTN
CRABP2
AFF3
F2RL2
GZMB
PTGS2
ISG15
COL15A1
CD83
HLA.DQA1
IDO1
BANK1
IGHD
AL590385.2
C5AR1
FYB1
APOC1
CLDN16
FCGR3A
CD163
CFD
MAL
C1QC
C1QB
ERBB4
C1QA
ADH1B
C7
PC_20
CXCL10
IFIT3
MT2A
IFIT2
CXCL11
TFPI2
MEDAG
TEX26.AS1
IFIT1
C1QA
C1QC
ISG15
GBP4
GBP1
IFIH1
KLRD1
CD83
GNLY
ITGAX
CCL4L2
G0S2
HOXB5
AL031316.1
SCGB2A1
IL1B
CCL3L3
AC025580.1
PTGDS
AC243829.4
HOXB.AS3
