# Supplementary figures and images for: Establishment of an ovarian cancer omentum metastasis-related prognostic model by integrated analysis of scRNA-seq and bulk RNA-seq
Source: J Ovarian Res. 2022 Nov 23;15:123. doi: 10.1186/s13048-022-01059-0 (PMC9686070; doi:10.1186/s13048-022-01059-0)

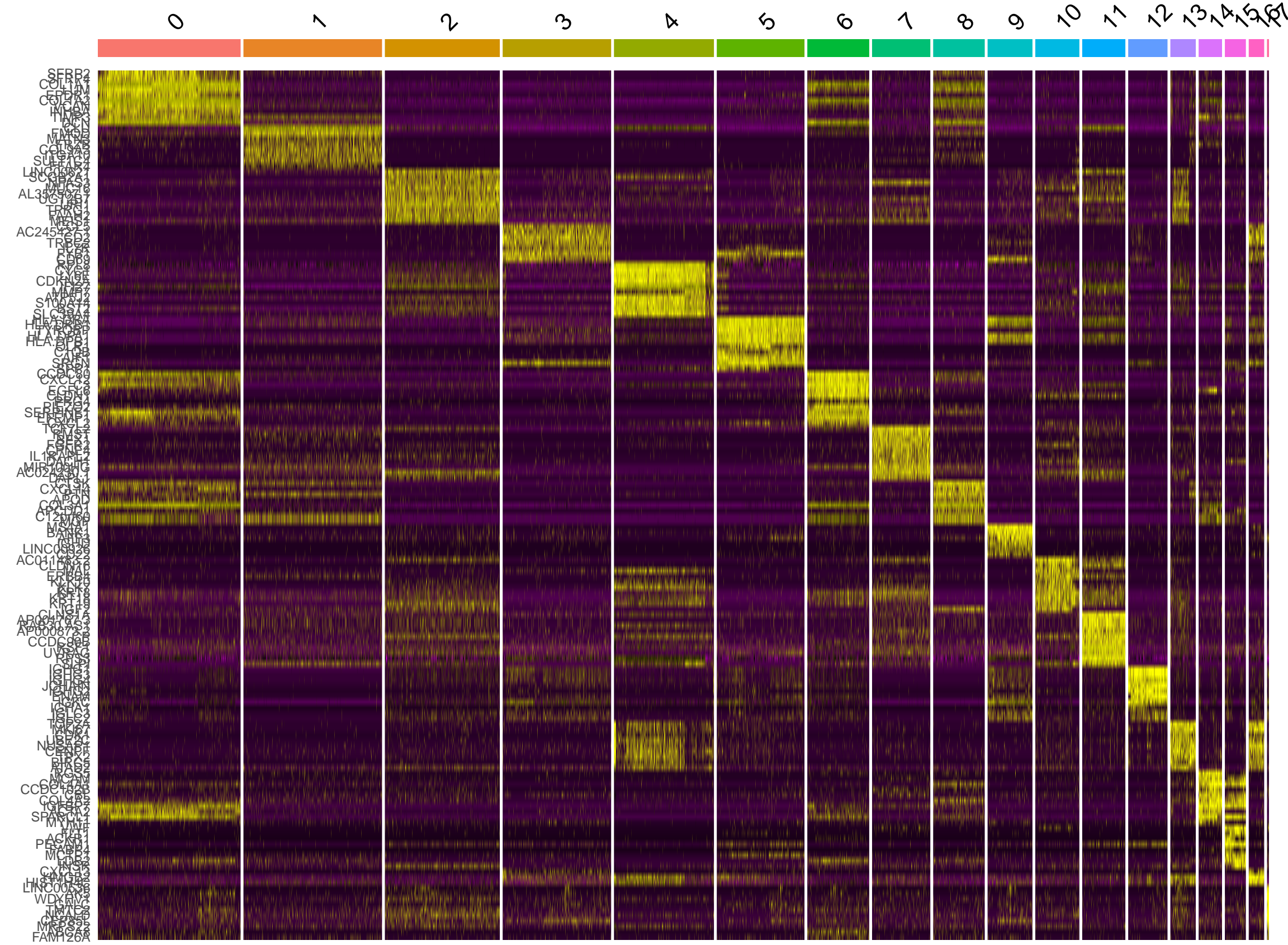

Supplement: Supplementary file 2 — Additional file 2. [file 13048_2022_1059_MOESM2_ESM.pdf]

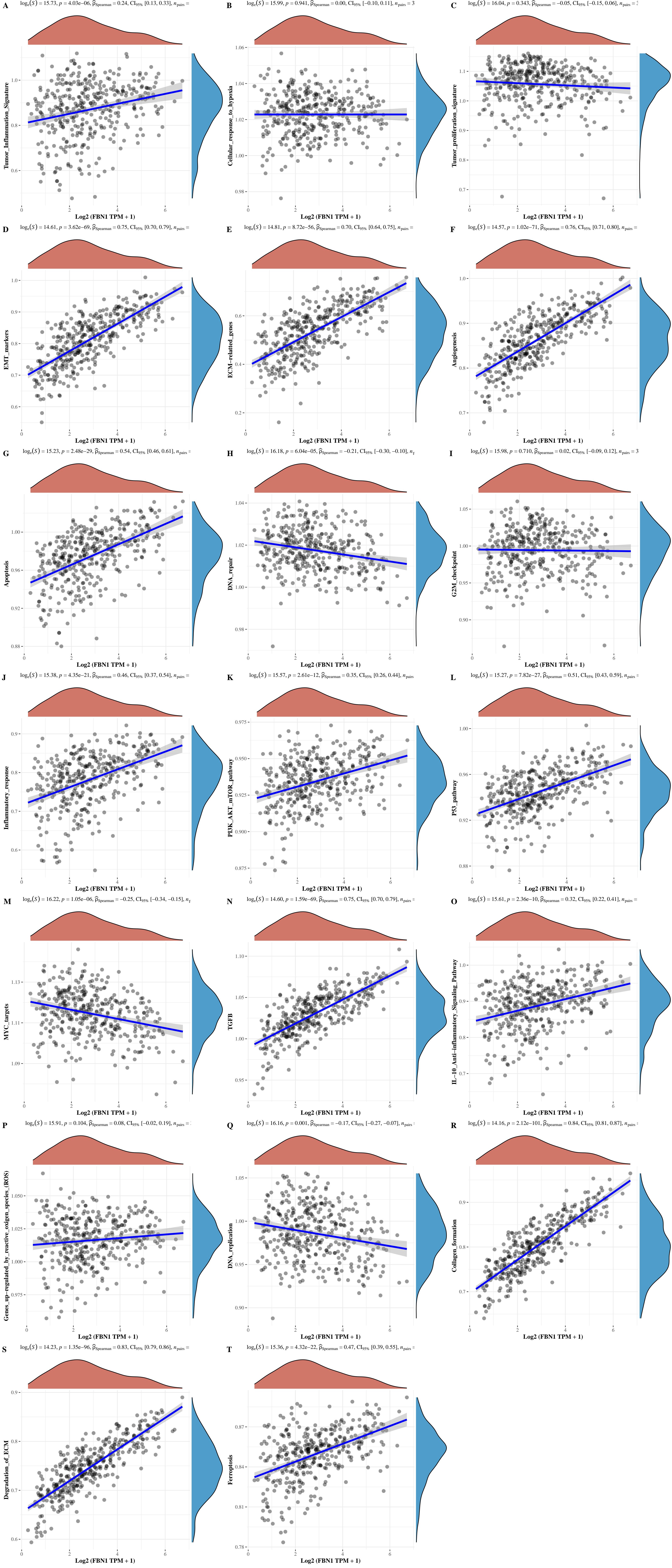

Supplement: Supplementary file 4 — Additional file 4. [file 13048_2022_1059_MOESM4_ESM.pdf]

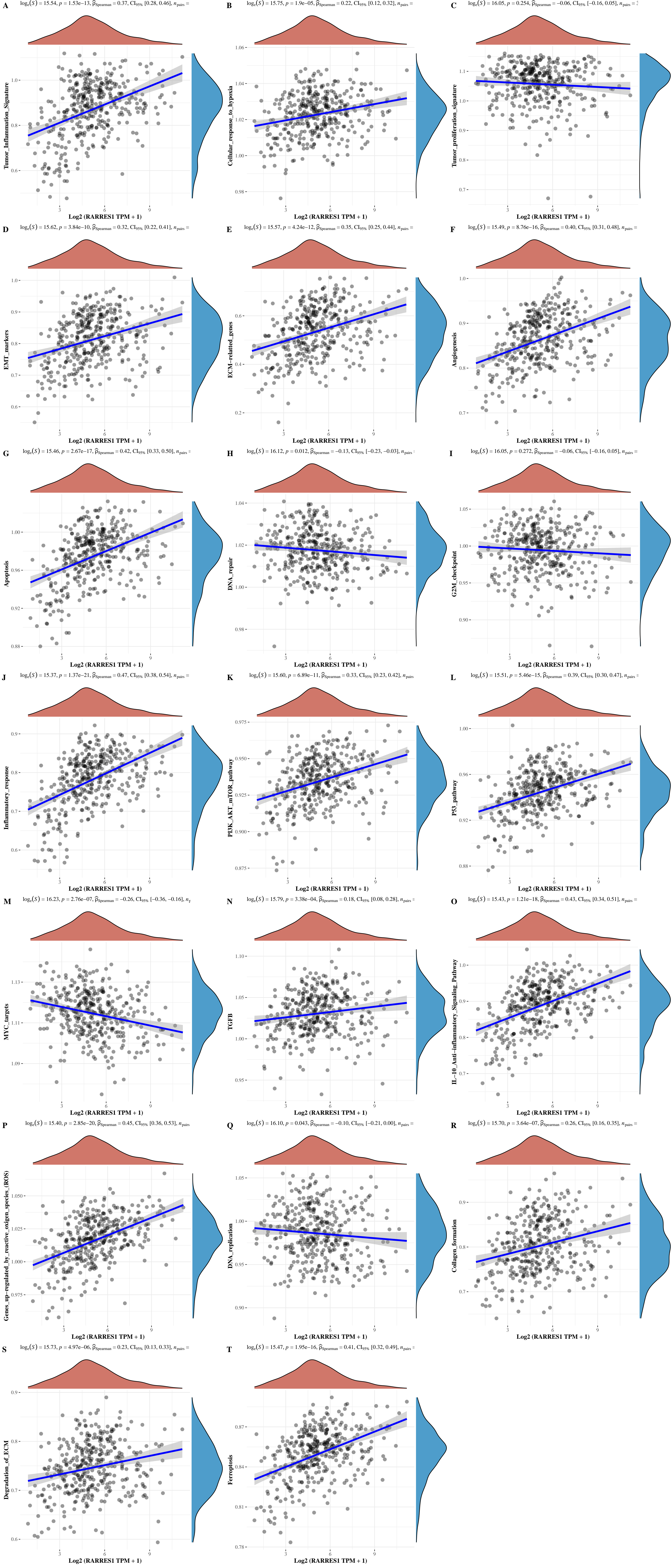

Supplement: Supplementary file 5 — Additional file 5. [file 13048_2022_1059_MOESM5_ESM.pdf]

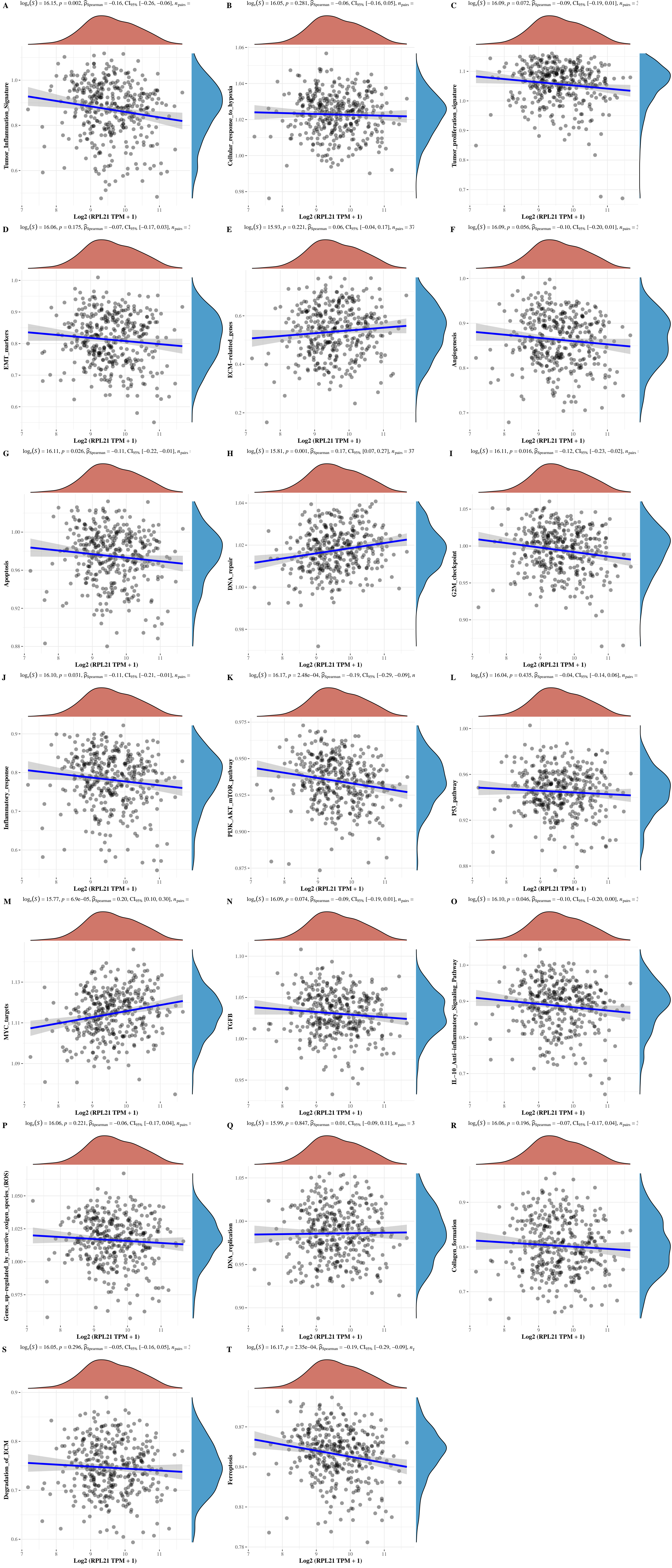

Supplement: Supplementary file 6 — Additional file 6. [file 13048_2022_1059_MOESM6_ESM.pdf]

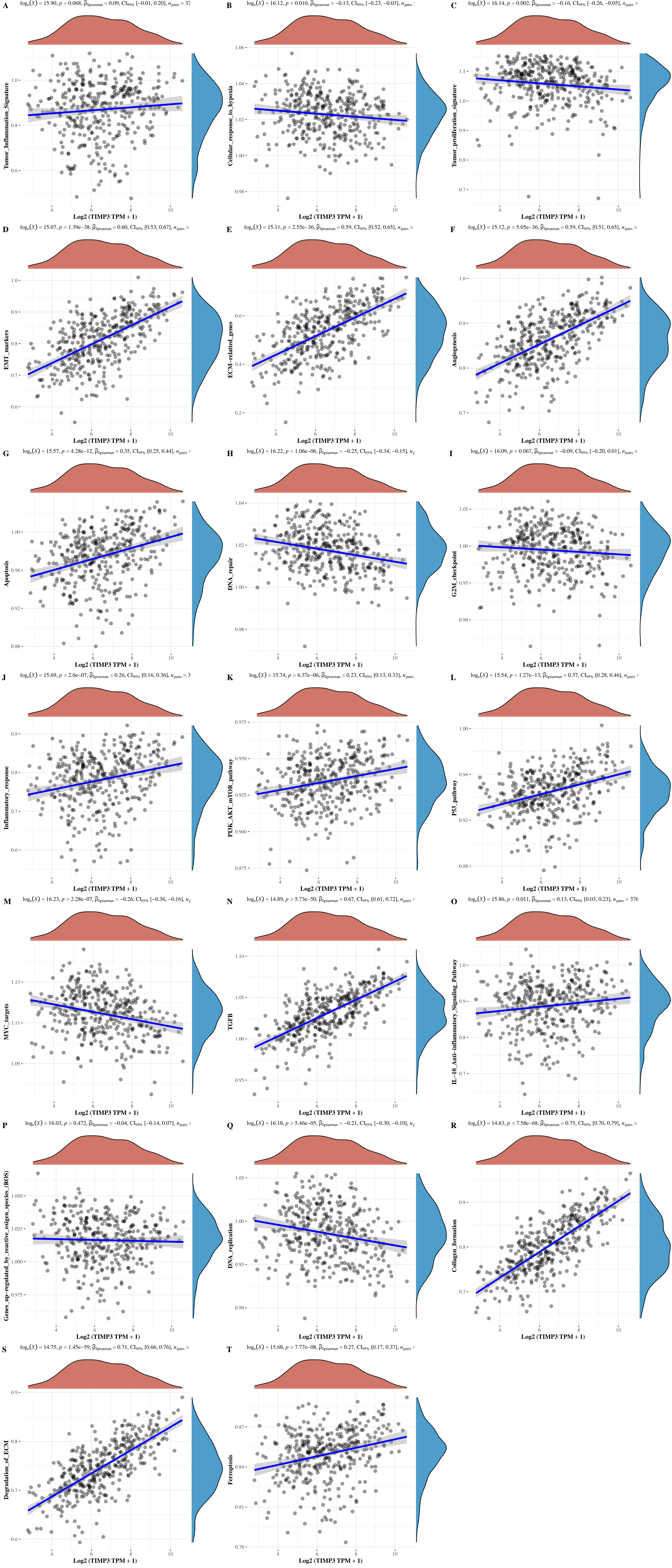

Supplement: Supplementary file 7 — Additional file 7. [file 13048_2022_1059_MOESM7_ESM.pdf]

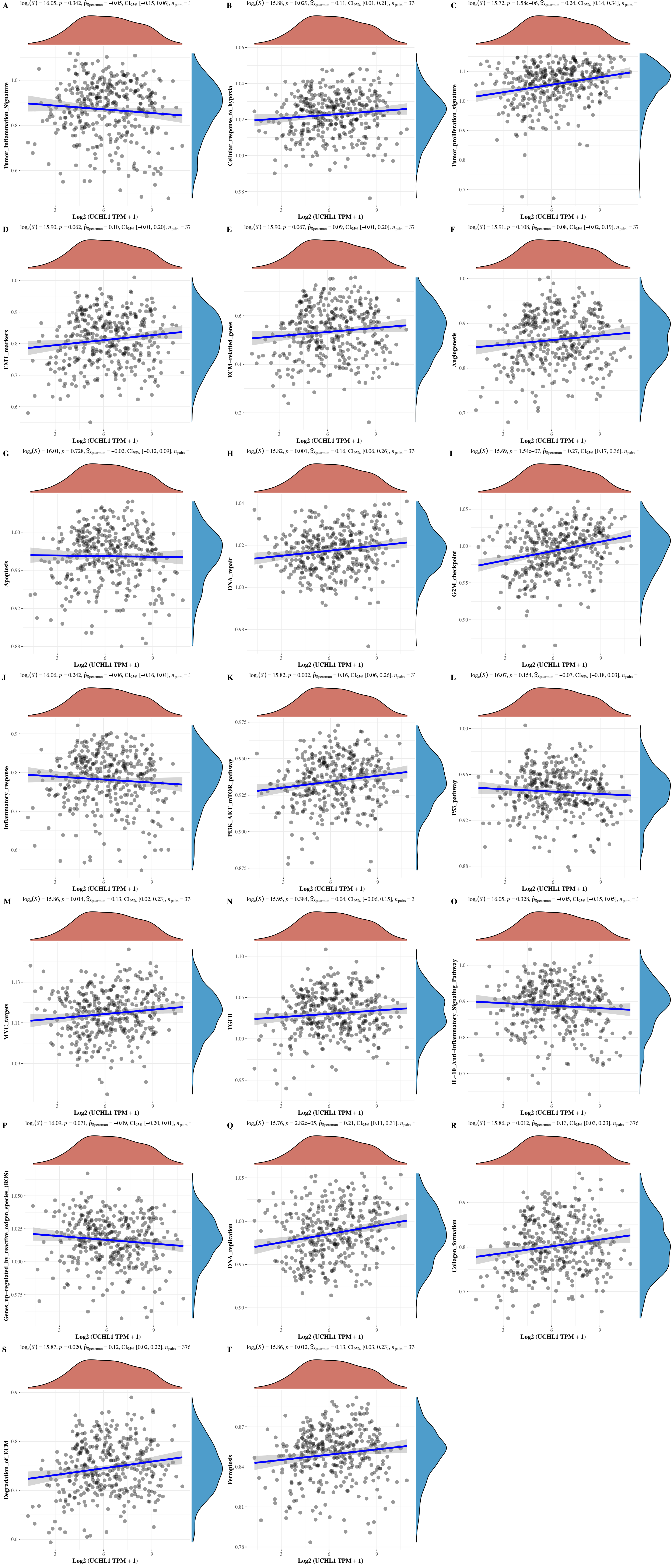

Supplement: Supplementary file 8 — Additional file 8. [file 13048_2022_1059_MOESM8_ESM.pdf]

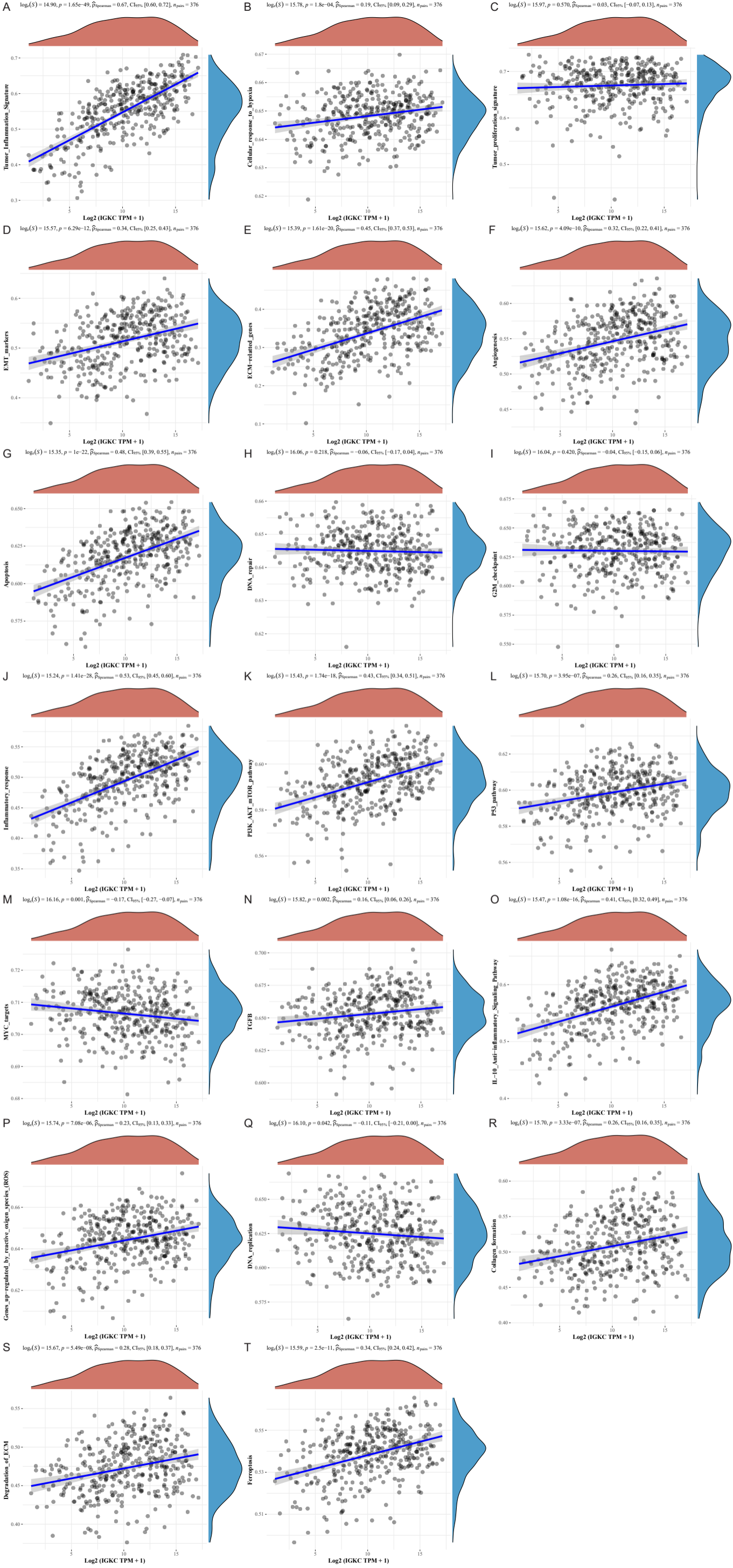

Supplement: Supplementary file 9 — Additional file 9. [file 13048_2022_1059_MOESM9_ESM.pdf]
